# The Non-Antibacterial Effects of Azithromycin and Other Macrolides on the Bronchial Epithelial Barrier and Cellular Differentiation

**DOI:** 10.3390/ijms26052287

**Published:** 2025-03-04

**Authors:** Arni Asbjarnarson, Jon Petur Joelsson, Fridrik R. Gardarsson, Snaevar Sigurdsson, Michael J. Parnham, Jennifer A. Kricker, Thorarinn Gudjonsson

**Affiliations:** 1 School of Health Sciences, University of Iceland, 101 Reykjavík, Iceland; 2 EpiEndo Pharmaceuticals, 102 Reykjavík, Icelandjk@epiendo.com (J.A.K.); 3 Department of Laboratory Hematology, Landspítali-University Hospital, 101 Reykjavík, Iceland

**Keywords:** macrolide, azithromycin, respiratory epithelial barrier, gene expression, EMT, air-liquid interface

## Abstract

The respiratory epithelium maintains the barrier against inhaled harmful agents. When barrier failure occurs, as in several respiratory diseases, acute or chronic inflammation leading to destructive effects and exacerbations can occur. Macrolides are used to treat a spectrum of infections but are also known for off-label use. Some macrolides, particularly azithromycin (AZM), reduce exacerbations in chronic obstructive pulmonary disease (COPD), whereby its efficacy is thought to be due to its effects on inflammation and oxidative stress. In vitro data indicate that AZM reduces epithelial barrier failure, evidenced by increased transepithelial electrical resistance (TEER). Here, we compared the effects of macrolides on differentiation and barrier integrity in VA10 cells, a bronchial epithelial cell line for 14 and 21 days. Erythromycin, clarithromycin, roxithromycin, AZM, solithromycin, and tobramycin (an aminoglycoside) were analyzed using RNA sequencing, barrier integrity assays, and immunostaining to evaluate effects on the epithelium. All macrolides affected the gene expression of pathways involved in epithelial-to-mesenchymal transition, metabolism, and immunomodulation. Treatment with AZM, clarithromycin, and erythromycin raised TEER and induced phospholipid retention. AZM treatment was distinct in terms of enhancement of the epithelial barrier, retention of phospholipids, vesicle build-up, and its effect on gene sets related to keratinocyte differentiation and establishment of skin barrier.

## 1. Introduction

The respiratory epithelium is continuously exposed to hazardous airborne infectious agents (viruses and bacteria) and toxic materials such as cigarette smoke and other particulate matter. Due to its frontline position, the respiratory epithelium is an important barrier against these external agents. Failure of the epithelial barrier allows agents to invade the sub-epithelial stroma and evoke an inflammatory response [1]. Chronic inflammation due to epithelial barrier failure disrupts homeostasis in airway diseases such as COPD, pulmonary fibrosis, and cystic fibrosis (CF) [2], whereby this disruption leads to progressive exacerbation of these diseases. The importance of the respiratory epithelial barrier is increasingly being recognized [1,3,4]. In COPD, the mucociliary clearance is dysfunctional and an epithelial-to-mesenchymal transition (EMT) phenotype is prominent, resulting in barrier failure [5]. The pseudostratified mucociliary epithelium can be reconstructed in vitro using bronchial-derived basal epithelial cells cultured under air-liquid interface (ALI) conditions [6], and ALI cultures are convenient cell culture models to test the efficacy of drugs that may affect the respiratory epithelium [7].

Macrolides are a class of antibacterial drugs widely used in the treatment of respiratory diseases; the name stems from their macrocyclical lactone ring (cyclical ester) [8,9]. Macrolides are often classified clinically by the number of members (the number of atoms making up the lactone ring structure) they contain. The most commonly clinically used macrolide antibiotics are 14–16-membered with one or more side chains attached to the lactone ring, the deoxysugars cladinose and desosamine being the most common [10]. The first macrolide discovered was erythromycin (ERY) isolated from *Saccharopolyspora erythrea* in 1952 [11,12]. Later macrolides are chemical modifications of ERY and are therefore referred to as second- and third-generation macrolides. These modifications resulted in longer half-lives and better tissue distribution with similar or better antibacterial activity. Commonly, macrolide antibiotics are used for their broad range of effects on Gram-positive bacteria. In recent years, macrolides have been shown to exert positive clinical effects on the host as well as on the clearance of bacteria. This has led to further interest in the effect of macrolides on eukaryotes. A variety of effects have been reported, including immunomodulatory effects, epidermal differentiation, lipid remodeling, inhibition of mucus secretion, and barrier enhancement [13,14,15,16].

As mentioned, second-generation macrolides are derivatives of ERY and thus slightly differ structurally from each other. The structural difference in azithromycin (AZM) from ERY lies in a methyl-substituted nitrogen in the now 15-membered ring. This change resulted in better distribution and a longer elimination half-life than ERY [17,18]. Clarithromycin (CLARI), also known as 6-*O*-methylerythromycin, has higher tissue distribution than ERY (extremely high in lung) [19]. Roxithromycin (ROXI; 9-[O[2-methoxyethoxy)methyl]oxime]-erythromycin), a 14-membered semisynthetic macrolide, was modified from ERY to combat inactivation in acidic gastric medium. It was shown to have the typical antibacterial activity of 14-membered macrolides [20]. Solithromycin (SOLI) is a fluoroketolide, where the cladinose was removed from ERY and a fluorine and a keto group added [21]. The macrolide antibiotic mode of action involves binding to the ribosomal 50s subunit of bacteria, inhibiting protein biosynthesis, whereby the hydrogen bond from desosamine binds to 23s rRNA [22]. Aminoglycosides act by binding to 16s rRNA of the 30s ribosome [23,24]. Tobramycin (TOBRA; nebramycin factor 6) was also derived from a bacterium, *Streptomyces tenebrarius*, and is used in the treatment of various infections (Gram-negative). It is a smaller molecule than the macrolides, with two amino sugar rings attached to a central cyclohexane ring. TOBRA shares a similar mechanism of action with macrolides, as they are all inhibitors of ribosomal protein synthesis, but TOBRA inhibits the formation of the 70s ribosomal subunit. The similarities do not end there as TOBRA is also used in respiratory infections and in patients with CF [25,26].

Immunomodulatory effects of macrolides were first reported as an in vitro effect of ERY treatment, where IL-6 production was significantly increased in monocytes isolated from healthy adult donors and stimulated with lipopolysaccharide (LPS) [27]. Macrolides were also shown to have a pronounced effect on disease prognosis in diffuse panbronchiolitis [28]. ERY was also shown to promote monocyte-to-macrophage differentiation [29] but to inhibit IL-6 release from cultured human bronchial epithelial cells [30]. Other macrolides were later shown to have immunomodulatory effects [15]; CLARI, for example, suppressed IL-1a, IL-1b, IL1ra, GM-CSF, TNF-a, IL-6 and induced IL-10 in LPS-stimulated monocytes [31]. AZM was later shown to suppress the production of the same cytokines as CLARI in LPS and Pansorbin-stimulated monocytes [32]. ROXI, like other macrolides, has been shown to have non-antimicrobial effects. It attenuates airway inflammation and the expression of receptors of advanced glycation end products (RAGE) and calprotectin in ovalbumin-challenged Brown Norway Rats (neutrophilic asthma model) [33]. ROXI has been reported to suppress the remodeling of the airway in ovalbumin-challenged rats (chronic asthma by induction). ROXI also modulated CAV-1 expression and phospho-p42/p44MAPK [34]. SOLI has been shown to attenuate IL-13 induction of goblet cell hyperplasia (*MUC5AC*, *CLCA1*, and *ANO1*). Inhibition of *CLCA1* and *ANO1* attenuates *MUC5AC* expression but did not have effects on phosphorylation of STAT6 and ERK [35]. SOLI suppresses MUC5AC in LPS-challenged respiratory epithelial cells [36]. TOBRA has also been shown to inhibit MUC5AC and suppress NFκB through p38 MAPK, as shown after LPS stimulation of the respiratory epithelial cell line NCI-H292 [37].

Due to the cationic amphiphilic properties of macrolides, they easily penetrate the plasma membrane/bilayer [38,39]. AZM is known to affect endocytosis, a suggested mechanism is by binding to lipids and thus affecting membrane organization [39]. The binding of AZM to lipids has been proposed as a mechanism for lipid retention as the access of phospholipase A1 to the lipids is decreased, in turn inhibiting lipid degradation [40]. In the VA10 ALI model used herein, AZM has been reported to increase vesicle formation (multivesicular bodies, lamellar bodies) and phospholipid accumulation, and has been shown to bind to lipids [41].

We and others have previously shown that AZM enhances the bronchial epithelial barrier in vitro [41,42,43,44,45]. Furthermore, we have shown that AZM treatment prevents barrier failure when bronchial epithelial cells are treated with culture medium or rhamnolipids derived from *Pseudomonas aeruginosa*, a bacterium commonly involved in lung diseases such as CF [43]. Due to all of the beneficial effects mentioned here, there is a clear need for the advancement of understanding in what manner and through which pathways AZM and other macrolides elicit their beneficial effects in combatting respiratory diseases. With increased use of AZM, the risk of resistant bacteria rises [46,47]. Several groups have recognized this need and have been generating novel 12- and 15-membered non-antibiotic macrolides [48,49,50,51].

The present study aims to deepen the knowledge of the effect of macrolides on the respiratory epithelia and to shed light on the structural-response relationship of macrolides. We present a comparison of the effects of the macrolides AZM, CLARI, ERY, ROXI, SOLI, and the aminoglycoside, TOBRA, on the VA10 bronchial cell line in an air-liquid interface model. We explore the effect of these molecules on differentiating ALI cultures on days 14 and 21. We analyze RNA sequencing data and the differences between treatments on cell-cell junctions and epithelial barrier integrity.

## 2. Results

### 2.1. Azithromycin Enhances the Bronchial Epithelial Barrier Function More than Other Macrolides

**Figure 1:**
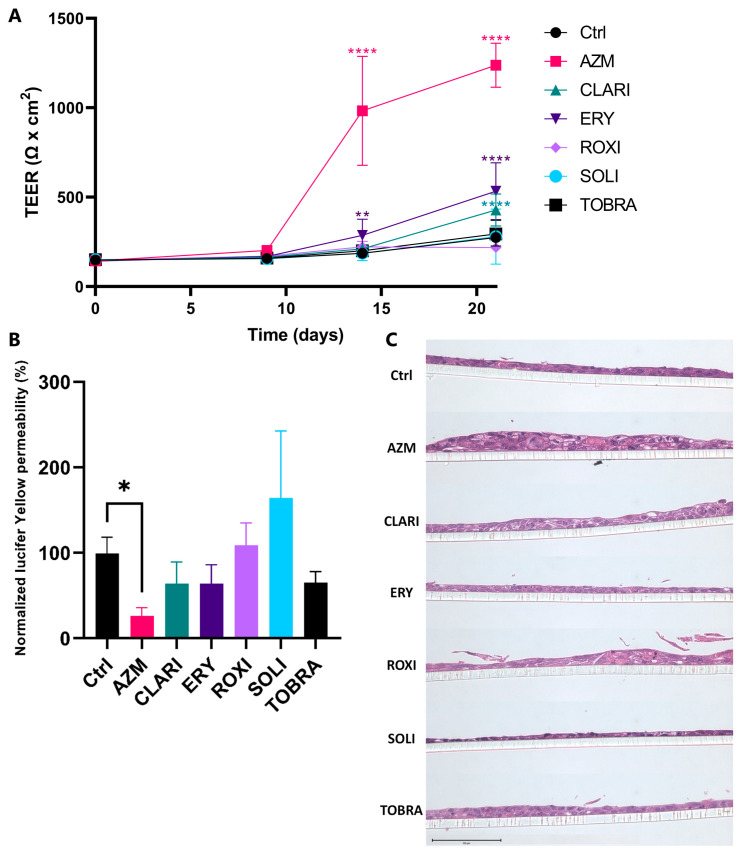
AZM treatment increases barrier function to a higher level than other macrolides. (**A**) AZM enhances TEER to a greater extent than other macrolides. VA10 cells were treated with 35 μM of the antibiotics over 21 days of ALI culture. AZM treatment increased TEER values significantly on days 14 and 21, compared to the control. CLARI and ERY treatment also increased TEER significantly, albeit not as much as AZM. Two-way ANOVA used for statistical analysis (** *p* < 0.005; **** *p* < 0.0001). *n* = 6. (**B**) AZM reduces the paracellular flux between the bronchial epithelial cells in ALI culture. Apical to basolateral Lucifer yellow permeability was measured on day 21 of ALI culture. AZM was the only antibiotic that significantly decreased Lucifer yellow permeability. Lucifer yellow was administered apically on day 21 and incubated for 2 h at 37 °C and 5% CO_2_. Basal liquid was then collected and measured using a microplate reader (excitation 485 nm/emission 535 nm). One-way ANOVA used for statistical analysis (* *p* < 0.05). *n* = 3. (**C**) AZM treatment of VA10 cells results in increased thickness of cell layers. H&E stained samples of ALI cultures on day 21 showing thickness and formation of vacuoles. ALI cultures were fixed in PFA then paraffin-embedded and cross-sectioned followed by an H&E staining. Scale bar = 100 μM.

The bronchial epithelial cell line VA10 was cultured under ALI conditions and treated with macrolides each at an equimolar concentration of 35 μM for three weeks. Control cells showed a slight increase in TEER from day 14 to day 21. Cells treated with AZM showed the highest TEER of all macrolide treatments with a steep increase from day 9, reflecting decreased epithelial permeability. ERY and CLARI treatments both significantly increased TEER from day 9 and 14, respectively, compared to the control (Figure 1A). Paracellular flux was measured at the end of the culture period (day 21). The lowest paracellular permeability was measured in AZM-treated cultures, being significantly different from controls. ERY, CLARI, and TOBRA treatments also tended to decrease paracellular flux, but this was not significant when compared to the control (Figure 1B).

Histological analysis showed an increase in thickness of epithelial cell layers when treated with AZM. ROXI and TOBRA also led to a moderate increase in thickness despite not significantly affecting TEER (Figure 1C). It is of note that viability assays showed no significant effect of treatments on viability (Supplementary Figure S1).

### 2.2. AZM Affects the Expression and Organization of Factors Participating in Cell Junctions and Tight Junctions

**Figure 2:**
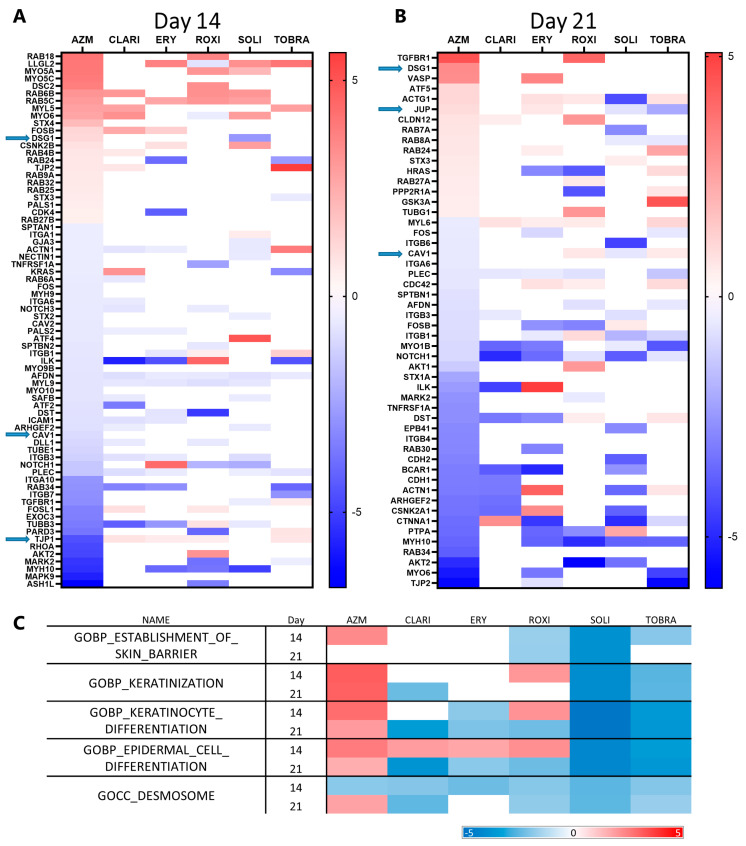
Only AZM enhances expression of gene sets related to epidermal and keratinocyte differentiation. (**A**) Heat map showing significantly upregulated focal adhesion, tight junction, gap junction, adherens junction, desmosomal, and hemidesmosomal genes on day 14 in ALI culture. Blue arrows note genes of interest that are uniquely expressed in AZM-treated cells, genes that were inspected at the protein level. Genes with a *p*-value higher than 0.05 and a beta value lower than 0.5 are shown as white. (**B**) Heat map showing significantly upregulated focal adhesion, tight junction, gap junction, adherens junction, desmosomal, and hemidesmosomal genes on day 21 in ALI culture. Blue arrows note genes of interest, that are uniquely expressed in AZM-treated cells, genes that were inspected at the protein level. Genes with a *p*-value higher than 0.05 and a beta value lower than 0.5 are shown as white. (**C**) AZM enhances expression related to epidermal and keratinocyte differentiation. Heat map of select GSEA gene sets. The table shows the GSEA GO biological process normalized enrichment scores for gene sets related to epidermal and keratinocyte differentiation. AZM treatment causes positive enrichment of those gene sets compared to controls, while the other treatments have a mostly negative or negligible effect on them. Enrichment values are a comparison to control by enrichment analysis. Data shown as enrichment differences with treatment of antibiotics at 14 and 21 days. Uncolored are not significant (*p*-value > 0.05). Red color represents increased enrichment relative to control and blue represents negative enrichment relative to control.

In our effort to explain the increased barrier function of AZM-treated ALI cultures, significantly upregulated focal adhesion, tight junctions (cell surface receptors, kinase signaling, actinins, and catenins), gap junctions, adherens junctions, desmosomes, and hemidesmosome genes were highlighted in volcano plots (including only one transcript per gene, the one with the lowest *p*-value) (Supplementary Figure S2). Genes that were significantly up- or downregulated more than two-fold were then compared across all treatments at both time points (Figure 2A,B). The most prominent effects of AZM treatment were the upregulation of *DSG1*, *DSC2*, *JUP*, and *CLDN12* (integral protein of the tight junction strands). Other adhesion-associated genes affected by AZM treatment include the downregulation of *TJP1*, *CAV1*, and *CAV2*. Among the downregulated genes, some were commonly downregulated across almost all treatments, such as *NOTCH1* and *PLEC*. Figure 2C provides an overview of the differences between AZM and other macrolides with regard to epidermal differentiation and keratinization seen using GSEA. On day 21, AZM is the only treatment that positively enriches the gene ontology biology process (GOPB) ‘establishment of skin barrier’. In reference to the gene groups ‘keratinization’, ‘keratinocyte differentiation’, and ‘epidermal cell differentiation’, AZM was the only treatment to positively enrich them at both time points.

**Figure 3:**
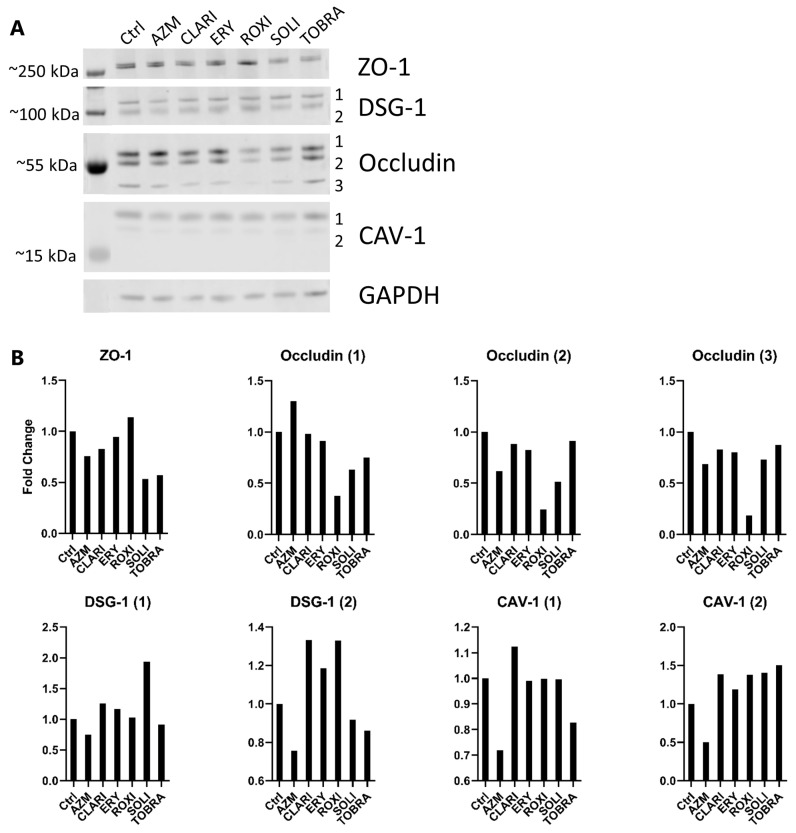
AZM has effects on occludin and CAV-1 protein expression. (**A**) AZM treatment caused lowered ZO-1, DSG-1, and CAV-1 protein levels. Occludin isoforms were either increased or decreased, respectively. Cell cultures were treated for 21 days in ALI condition before lysing and western blotting. Blots were probed for ZO-1, occludin, CAV-1, and DSG1. Probing revealed two separately sized bands for DSG-1 and CAV-1, and three for occludin. (**B**) Quantification of protein bands shown in 3A. GAPDH was used as a loading control and quantifications were performed using FIJI for area under curve analysis.

**Figure 4:**
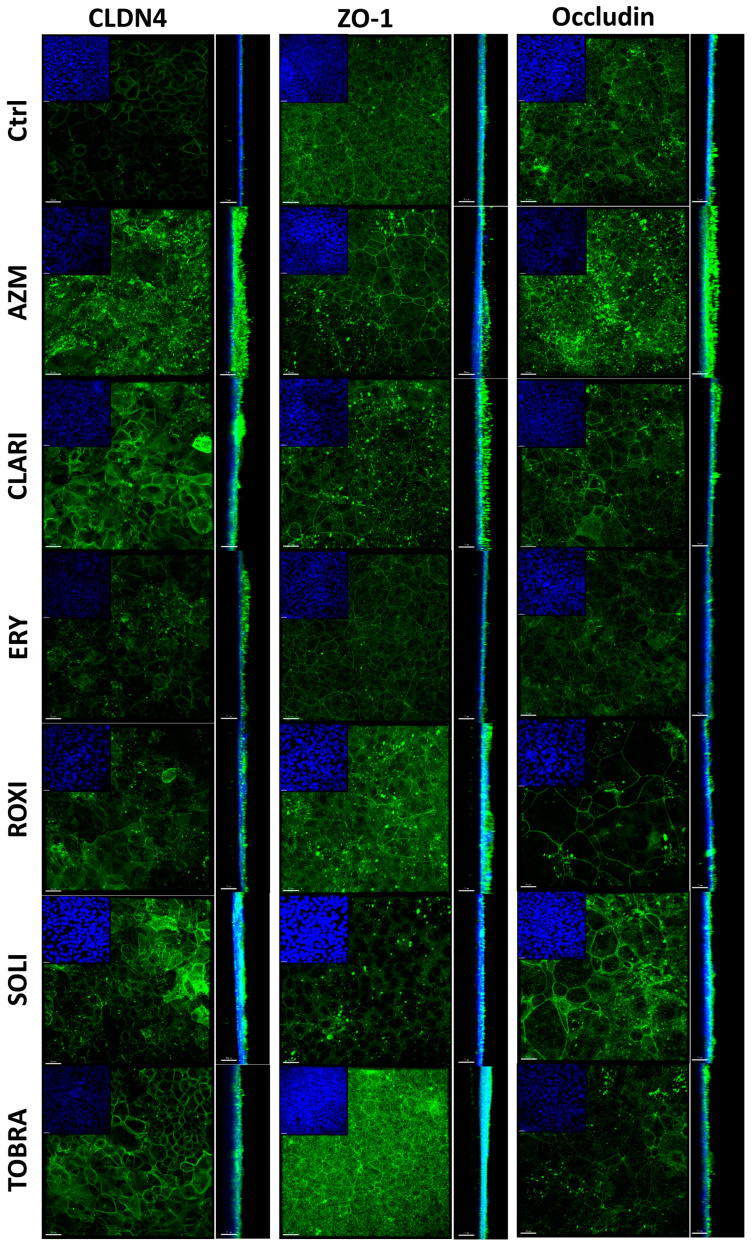
Macrolides affect tight junction protein localization. AZM specifically increases apical and cell-cell junction localization. ALI cultures were fixed in an equal parts acetone: methanol mixture before permeabilization and staining of the tight junction proteins, claudin 4, ZO-1, and occludin. Tight junction protein (green) and DAPI-stained nuclei (blue). Orthogonal view presented next to each image. Scale bar = 20 μM.

RNA sequencing data show that *TJP1* (ZO-1) was downregulated at the gene level with AZM treatment (Figure 2A), which agrees with the Western blots (Figure 3) and the immunostainings (Figure 4).

Western blotting confirmed that there were three isoforms of occludin of approximately 59, 54, and 52 kDa. AZM increased the protein expression of the largest isoform of occludin but lowered that of the others. Other treatments did not affect expression notably but had a tendency to decrease expression. ROXI caused the greatest decrease, followed by SOLI and TOBRA. DSG-1 protein expression was decreased by AZM for both the presumed precursor (DSG-1(1)) and mature (DSG-1(2)) forms. CLARI, ERY, and ROXI all increased the mature form considerably. Interestingly, only SOLI caused a considerable increase in the precursor form. When blotting for CAV-1, two isoforms of CAV-1 were observed: (1) 20 and (2) 17 kDa. Only AZM lowered the protein levels of both.

**Figure 5:**
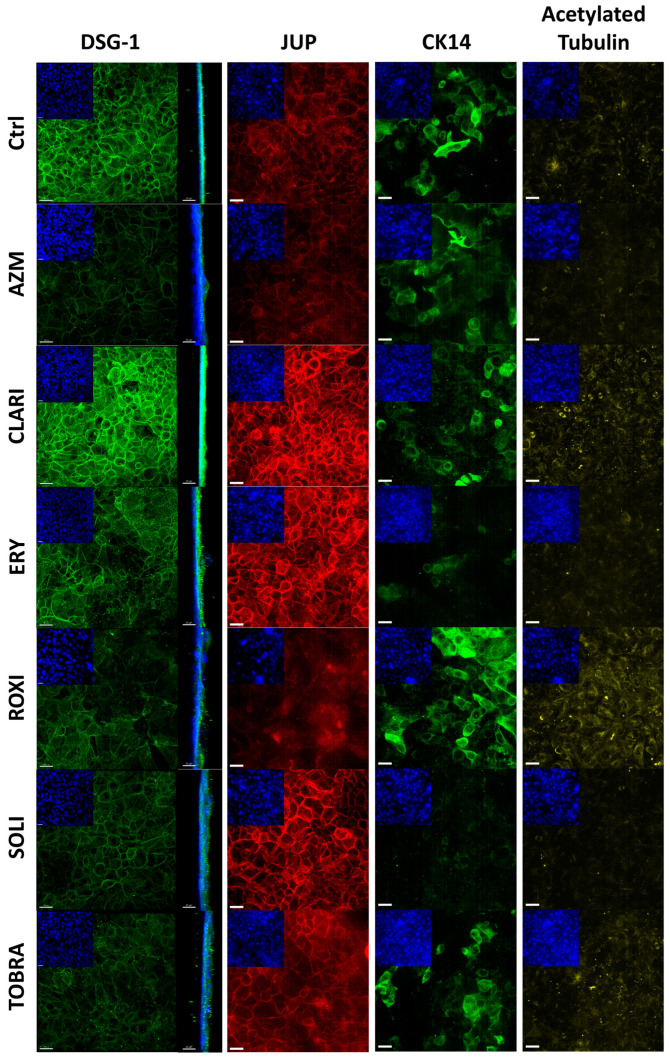
AZM causes apical localization of desmosomal protein and increased differentiation. ALI cultures were fixed in an equal parts acetone: methanol mixture before permeabilization and staining of the desmosomal junction proteins, DSG-1 and JUP, and differentiation markers CK14 and acetylated tubulin. CK14 is a basal cell marker and acetylated tubulin is needed for cilia and cell polarization. Location of DSG-1 in AZM-treated cells is restricted to the cell membrane and apical position; see orthogonal projection. DSG-1 and CK14 (green), JUP (red), acetylated tubulin (yellow), and DAPI-stained nuclei (blue). Scale bar = 20 μM.

We took a closer look at the tight junction proteins previously shown to be affected by AZM [42,43] and followed relevant cell-cell junction genes seen in our sequencing data. ALI cultures treated with macrolides for 21 days were fixed and immunostained for tight junction proteins, (Figure 4) desmosome, and differentiation markers (Figure 5). AZM treatment resulted in punctated expression of claudin-4 and occludin. CLARI and TOBRA induced clear membrane staining of claudin-4, whereas ERY, SOLI, and ROXI treatment resulted in vague staining. The orthogonal view of the staining shows that tight junctions are present apically rather than basolaterally in the cell-cell junctions. Furthermore, the orthogonal view shows that AZM treatment resulted in a supra-apical concentration of occludin and claudin-4. ZO-1 is localized more at the apical cell-cell junction in AZM-treated cells than in the control samples, where the expression is more uniformly spread across the cell-cell junction. CLARI treatment also resulted in a similar distribution of occludin and claudin-4. Claudin-4 is seen apically in CLARI-treated cultures, but not to the same extent as in AZM-treated cultures. SOLI resulted in diffuse ZO-1 staining at the cell-cell junction and irregular staining of occludin. ROXI treatment resulted in irregular occludin expression.

After treatment with AZM, DSG-1 was mostly localized at the apical part of the cell-cell junction. The effect on JUP was similar to that of DSG-1. Treatment with CLARI, ERY, and SOLI increased JUP expression, but it was lowered after AZM and ROXI treatment.

CK14 is a basal cell marker, and on day 21 in ALI culture, some differences in CK14 expression were visible when comparing treatments, including a higher level of expression in ROXI-treated cells and low levels after SOLI treatment (Figure 5).

### 2.3. Gene Set Enrichment Analysis Indicates All Macrolides Have a General Effect on Metabolism and EMT

EMT pathways are considered a possible cause or consequence of respiratory epithelial barrier dysfunction in COPD [5,52]. AZM has been shown to improve lung function in both COPD [53] and CF [54,55] and to increase barrier resistance in vitro [42,43]. We thus decided to investigate the effect of macrolides on EMT pathways and gene expression in our model.

**Figure 6:**
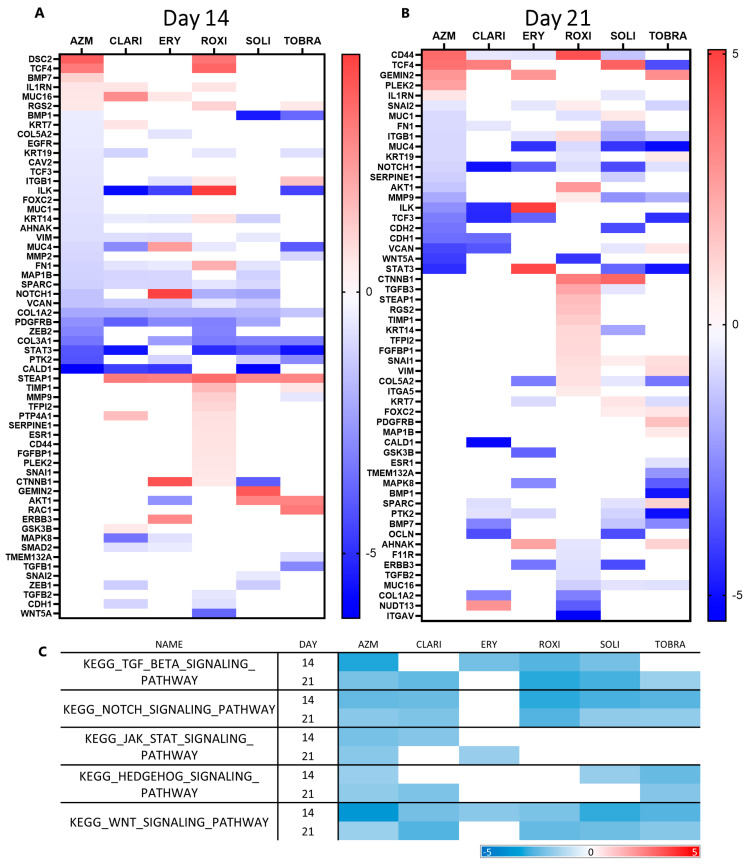
Macrolides downregulate pathways associated with EMT. (**A**) Heat map of a variety of EMT-related genes where expression compared to control was affected by macrolide treatment on day 14 in ALI culture. (**B**) Heat map of a variety of EMT-related genes where expression compared to control was affected by macrolide treatment on day 21 in ALI culture. (**C**) AZM negatively enriches more EMT-related pathways than other macrolides. Macrolides downregulate pathways associated with EMT. Heat map showing GSEA results for KEGG pathways. Red color represents increased enrichment relative to control and blue represents negative enrichment relative to control.

EMT markers that had a *p*-value below 0.05 and a beta value above 0.5 were identified (Supplementary Figure S3). All genes affected were then compared across all treatments separately at each time point (Figure 6A,B). Of the EMT genes differentially expressed with treatment, most of them were downregulated by the macrolides, apart from ROXI, after which more than half of the affected genes were upregulated.

*TCF4* and *IL1RN*, which are frequently upregulated during EMT, were upregulated on both days 14 and 21 after AZM treatment. EMT-related genes that were downregulated with AZM treatment include *NOTCH1*, *VCAN*, (involved in differentiation and development), *ILK* (cell growth and proliferation, also ECM and adhesion), *ITGB1* (Integrin-mediated signaling), *KRT19* (Estrogen Receptor signaling), *FN1* (migration, motility, ECM adhesion), and *STAT3* (migration and motility). Notably, collagen turnover seems to be affected, as *COL1A2*, *COL3A1*, *COL5A2*, *MMP2*, and *MMP9* were all downregulated with AZM treatment.

When comparing heat maps showing GSEA enrichment of Kyoto Encyclopedia of Genes and Genomes (KEGG) pathways, there are clear similarities between the different macrolides. All compounds affected multiple EMT pathway enrichment negatively to an extent, but only AZM negatively enriched them all significantly at both time points (Figure 6C). TGFβ signaling, Notch signaling, JAK-STAT signaling, Hedgehog signaling, and WNT signaling pathways were all negatively enriched by AZM at both time points. CLARI treatment offers a similar effect on the presented gene set pathways, as all were at least negatively enriched at a single time point. ROXI, SOLI, and TOBRA did not affect JAK-STAT signaling. ERY is least like AZM when it comes to its effect on EMT, as only TGFβ, JAK-STAT, and WNT signaling were negatively enriched at a singular time point each.

### 2.4. Azithromycin Treatment Induces a Higher Build-Up of Phospholipids and Vesicles than Other Macrolides

**Figure 7:**
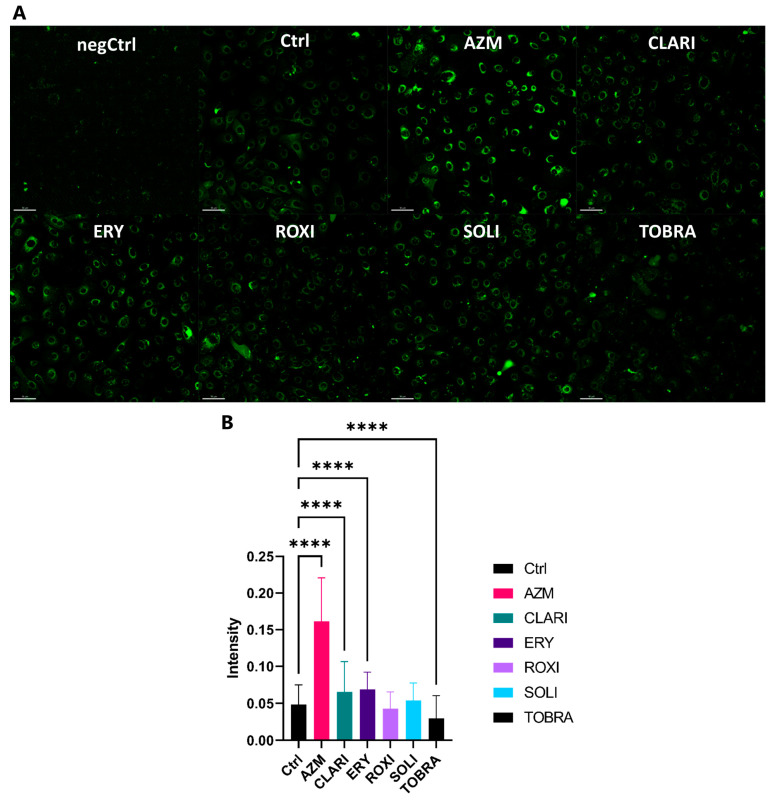
AZM treatment induces a higher build-up of phospholipids and vesicles than other macrolides. (**A**) LipidTOX assay images. VA10 cells in monolayer were treated with the antibiotics and HCS phospholipidosis LipidTOX. VA10 cells were treated with 35 μM of the various antibiotics and HCS phospholipidosis LipidTOX green for 72 h before fixing using formaldehyde and staining nuclei with DAPI. Cells were then imaged with Alexa Fluor 488 dye and DAPI filter sets. (**B**) LipidTOX assay, intensity quantification. Images from the LipidTOX assay were analyzed using a CellProfiler pipeline where each nucleus was established as a primary object and the secondary objects were defined as the LipidTOX signal. The intensity of that signal was then measured per cell. One-way ANOVA used for statistical analysis (**** *p* < 0.0001). *n* = 3.

**Figure 8:**
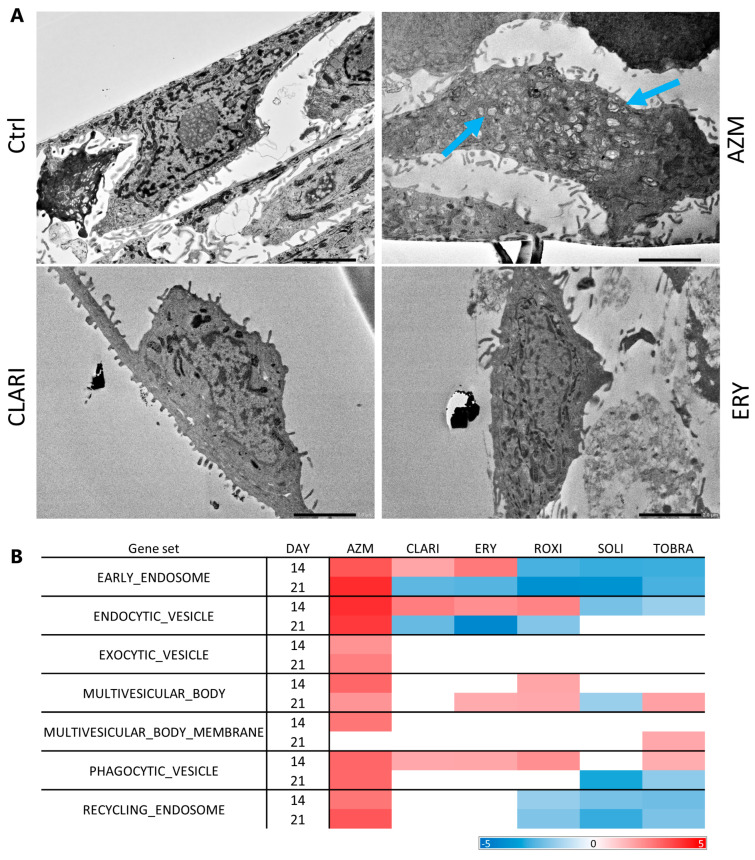
Multivesicular bodies are only prominently found in AZM-treated cells. (**A**) Transmission electron microscope images. ALI cultures were fixed and prepared for TEM. Blue arrows point out vesicles. Scale bar = 2.0 μM. (**B**) Table showing enrichment of GSEA from GO cellular components gene sets related to multivesicular bodies. Values highlighted in red are positively enriched and values highlighted in blue are negatively enriched. Unhighlighted values are not significant (*p*-value > 0.05).

Macrolides are cationic amphiphilic drugs that can bind phospholipids and inhibit their degradation, resulting in the accumulation of lipids within the cells [38,39,56]. Here, we analyzed the ability of macrolides to induce phospholipid retention in a monolayer culture of VA10 cells. Cells were treated for 3 days with macrolides (35 μM). Phospholipids were stained using LipidTOX, a probe designed to detect phospholipids, then imaged after fixation (Figure 7A). The intensity of the phospholipids per cell was quantified using a CellProfiler pipeline where average intensity per cell was measured [57]. The representative images and quantification (Figure 7B) show that AZM treatment had the most significant impact on increased phospholipid retention, three-fold that of the control. CLARI and ERY also increased phospholipid retention, but interestingly, treatment with these two did not seem to induce vesicle build-up. As clearly seen in transmission electron images of AZM-treated ALI cultures, only AZM treatment caused vesicle build-up, whereas CLARI and ERY did not show any effect (Figure 8A). RNA sequencing is in agreement with these images, as GSEA showed that AZM affected cellular component gene sets involved in vesicle formation to a greater extent than other macrolides (Figure 8B). CLARI and ERY were closest to AZM in the enrichment of gene sets but did not affect cellular component gene sets of ‘multivesicular body membrane’, and endosomal-related gene sets were negatively enriched on day 21.

### 2.5. RNA Sequencing Analyses Show That AZM Has a Distinct Expression Pattern Compared to Other Macrolides

**Figure 9:**
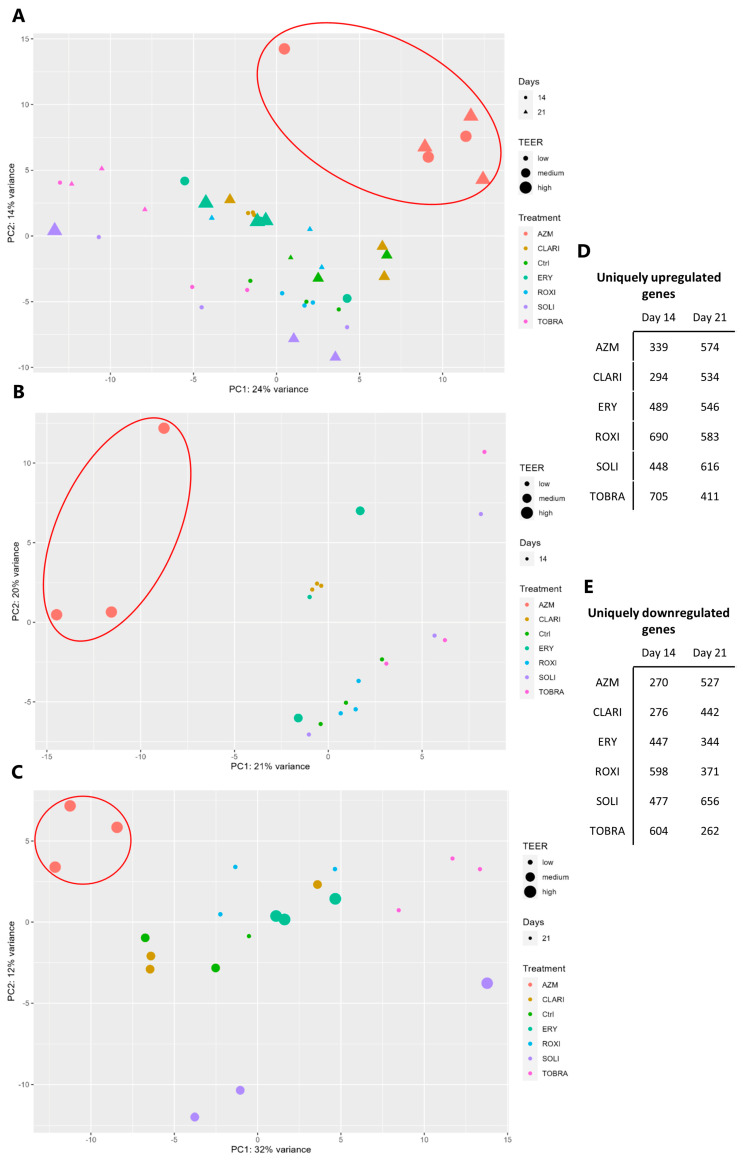
Principal component analysis shows an increase in AZM treatment clustering with longer treatment time. (**A**–**C**) Principal component analysis comparing samples from both time points (**A**), day 14 (**B**), and day 21 (**C**). Each treatment is represented with different colors and shapes are used to differentiate time points when both are plotted on the same graph. Circles depict samples from day 14 and triangles depict samples from day 21 (**A**). The size of symbols indicates the TEER value of the data point (low = 0–299; medium = 300–499; high = 500+). Red ellipses are used to show the clustering of AZM-treated samples, principal components successfully differentiated AZM-treated samples from the other compounds tested. Percentage of Variance is how much of the variance is explained by the principal component. Principle components 1 and 2 are plotted on the x and y axis. (**D**,**E**) Table showing uniquely affected genes with each treatment on days 14 (**D**) and 21 (**E**).

VA10 cells in ALI culture were treated with macrolides for 14 or 21 days before RNA isolation and sequencing. Principal component analysis (PCA) shows that on day 14, there was a clear effect of all the treatments on VA10 cell gene expression (Figure 9A,B). AZM-treated samples start to cluster together and are even more closely related on day 21 than on day 14. On day 21, the clustering of treatments is more pronounced (Figure 9C), indicating that either longer treatment time or only the differentiated cells are affected by AZM to a higher degree.

To compare the effects of macrolide treatment and measure the differences caused on gene expression, Venn diagrams were made from ranking gene lists normalized to the vehicle-treated control expression. Venn diagrams (Supplementary Figures S4 and S5) show the number of expressed genes that are common to or unique to each macrolide. A total of 339 genes (3.9774%) and 574 genes (7.2667%) were upregulated and unique to AZM on days 14 and 21, respectively (Figure 9D,E). Those genes that were common between AZM, CLARI, and ERY were reduced from 4225 (34.2%) on day 14 to 2814 (20.6%) on day 21 (Supplementary Figure S4). We also analyzed the number of downregulated genes. There was only a minor increase in the number of downregulated genes between days 14 and 21 in AZM-treated cells (Supplementary Figure S5). In contrast, there was a sharp increase in negatively regulated genes between days 14 and 21 in CLARI-treated cells. The genes that were only affected by AZM treatment were then further analyzed by performing an overrepresentation test, using PANTHER, for biological process and cellular component analysis (Supplementary Table S1). Interestingly, gene ontology (GO) families such as epidermis and skin development, as well as cornified envelope, were highly prominent, indicating that these pathways could be involved in the barrier formation in VA10 cells. Also, several GO families that were highly expressed were involved in vacuole formation that may be linked to lipid accumulation. Many of the GO families that were downregulated are involved in ribosomal biogenesis and protein synthesis.

The Venn diagrams also show that from day 14 to day 21, the effects of the drugs on gene expression become more specific, with the number of genes commonly upregulated or downregulated decreasing. The drugs that have the most drastic effects are the ones that have the highest number of unique up- or downregulated genes (Figure 9D,E).

**Figure 10:**
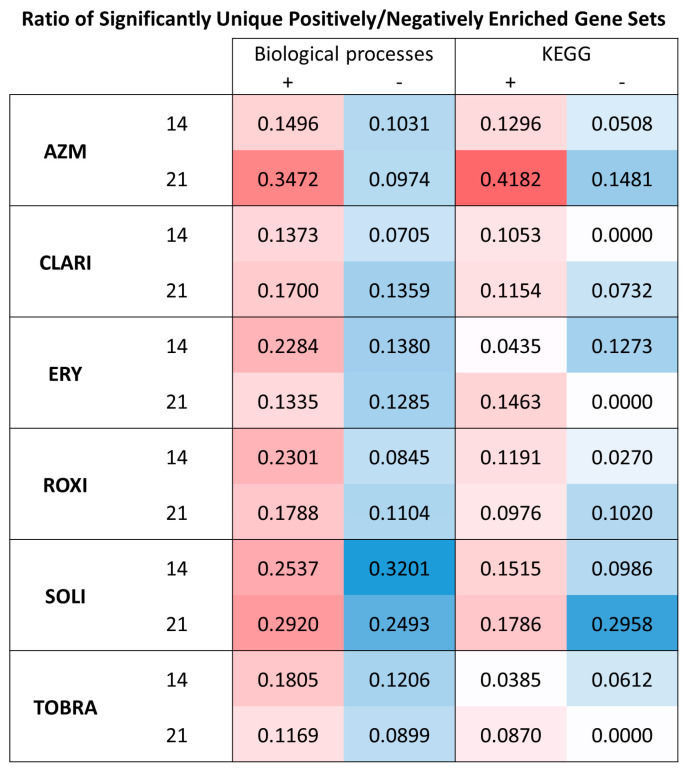
AZM causes the most unique positive enrichment of GO biological processes and KEGG gene sets. The figure shows the ratio of uniquely significantly enriched gene sets out of all enriched gene sets. All significant gene sets from GSEA of KEGG and BOBP were compared on both days. The ratio shown is the number of gene sets that were only enriched significantly by each variable divided by the total amount of significantly enriched gene sets.

Comparison of unique, significant positively, or negatively enriched GO biological processes shows that AZM treatment causes the highest ratio of uniquely positively enriched gene sets on day 21. There were 242 significant (nominal *p*-value of 0.05) GO biological process gene sets that were positively enriched with AZM treatment on day 21. This is considerably higher than the number seen with the other treatments. ROXI treatment, which positively enriched the second-highest number of gene sets, only did so for 106 unique GO biological processes. SOLI negatively affected by far the most unique GO biological processes, enriching 352 groups (Figure 10).

## 3. Discussion

In this study, we have explored the non-antibacterial effects of macrolides on bronchial epithelial cell differentiation under ALI conditions. The main findings are that AZM is superior to other macrolides in enhancing the epithelial barrier. The effects on epithelial barrier enhancement are evidenced by increased TEER and lowered paracellular flux. ERY and CLARI caused a moderate increase and decrease in TEER and paracellular flux, respectively. The cellular thickness was most prominent in AZM, CLARI, and ROXI-treated cells, though viability was not significantly affected by any treatment.

During COPD, cell polarization and differentiation are abnormal, and the barrier function of airway epithelium is compromised [52,58]. Thus, we used our model to assess the effectiveness of macrolide treatment on cell differentiation and barrier integrity of the airway epithelium.

Immunostainings for tight junction proteins showed an interesting pattern after treatment with AZM, with a supra-apical location of claudin-4, occludin, ZO-1, JUP, and DSG-1. This apical redistribution of tight junction proteins, particularly of ZO-1, has been shown to occur pathophysiologically to close paracellular spaces. For instance, on the one hand, enteropathogenic *Escherichia coli* displaces these junction proteins in gut epithelium [59], while in response to endogenous trefoil peptides, the tight junction proteins are returned to the apical surface of the gut epithelium [60]. Consequently, the protein redistribution with AZM is likely to contribute to barrier enhancement.

RNA sequencing showed that AZM indeed had a drastically different effect on gene expression when compared to the other macrolides tested. It is also of note that as the epithelium matures in culture, the number of commonly up- or downregulated genes is lowered. With time, PCA clustering becomes more specific with each treatment. Macrolide treatment showed more distinct clustering on day 21 than on day 14 (Figure 9B,C). All macrolides downregulated gene sets involved in EMT, but only AZM positively affected gene categories related to the differentiation of keratinocytes and epidermis. Arason et al. showed an upregulation of genes involved in late epidermal differentiation and barrier formation following AZM treatment in ALI cultures [41]. In this present study, we confirm these earlier findings with our results from AZM-treated ALI cultures, giving confidence to the results we present for the other drugs. These GO differentiation and barrier-promoting groups are specific to AZM-treated cells and are not upregulated following treatment with the other tested macrolides. This is further supported by the fact that when a PANTHER overrepresentation test was run using only genes upregulated by AZM treatment, the GO biological process categories that were positively enriched were related to skin and epidermal development (Supplementary Table S1). This suggests that genes involved in these biological processes may contribute to the barrier-enhancing effects of AZM on the epithelium.

At the molecular level, the barrier-enhancing events are most likely multifactorial including changes in the expression and organization of cell-cell junction proteins and cell signaling pathways. When comparing confocal images of tight junctional proteins to TEER measurements and paracellular flux assays, the fortification of barrier functions is not immediately clear on confocal images. The barrier function effects seen in samples treated with AZM might be caused by apical relocation of claudin-4 and occludin, as discussed above. The difference in the ratio of occludin isoforms is also a possibility. ZO-1 being localized to the apical cell-cell junction is possibly the most likely answer, as ZO-1 staining of tight junctions seems to be brighter at the cell-cell junction than for other conditions. Interestingly, it is not immediately clear why ERY treatment significantly increases TEER when comparing the tight junction staining to that of AZM and CLARI treatments, as ERY treatment does not exhibit the same distribution of ZO-1 and occludin, apart from being organized similarly at the lateral-apical cell-cell junction.

ZO-1 has been reported to form cytoplasmic condensate droplets that drive the polymerization of tight junction proteins to form a continuous junction linking cells tightly to each other and to the actin cytoskeleton in individual cells [61,62,63,64]. When looking at the function of ZO-1 in other tissues, for example in the gut, it is not necessary for barrier function but is important for mucosal repair [65]. Possible reasons for this barrier integrity difference might be traced to gene expression differences, leading to increased expression of barrier-related genes (keratinocytes, skin barrier, etc.). Other possible reasons include the downregulation of CAV-1 we observed with AZM, as CAV-1 interacts with tight junctions and affects both their expression and localization [66]. LPS-induced overexpression of CAV-1 at the mRNA and protein levels in lung endothelial cells contributed to an increase in permeability and a reduction in CAV-1 was also shown to decrease permeability [67,68,69]. We found both a decrease in CAV-1 at the RNA level as well as at the protein level in AZM-treated samples which might be a contributing factor to the decrease in permeability and increase in TEER.

DSG-1, a part of the E-cadherin superfamily, is one of the major desmosomal cadherins [70,71] and is strongly expressed in the epidermis, providing barrier function to the skin. It is also expressed in mucosal tissue such as the bronchial epithelium [72]. A significant decrease in E-cadherin has been reported in preterm-born children with bronchopulmonary dysplasia [73]. Conversely, treatment with inhaled corticosteroids with a long-acting β2-agonist significantly increases DSG-1 expression, potentially contributing to an improved bronchial epithelial barrier [73,74]. Several other studies have linked DSG-1 location and expression with epithelial barrier function [75,76,77]. In our desmosomal stainings, we show a more localized signal of DSG-1 and JUP in AZM-treated ALI cultures. Localization is more apparent at the apical side of the cell-cell junction and little is seen intracellularly when compared to other treatments. Oddly, DSG-1 protein levels in both the proposed precursor and mature forms are lowered with AZM treatment. However, gene expression of DSG-1 is increased with AZM treatment, and this disparity might be caused by post-transcriptional regulation or degradation.

With the data shown here on the tight junction and desmosome components, it is noteworthy that ZO-1, DSG-1, and JUP all seem to exhibit lowered protein levels with AZM treatment, but of all the tested molecules, it was the most efficient in decreasing paracellular flux and increasing TEER. The rate-limiting step of paracellular flux is the maintenance of the intracellular tight junction, so the differences seen in the Lucifer yellow assay should be explainable in terms of factors regulating the tight junctions [78]. We therefore propose that the differential effects of macrolides seen on the barrier may be down to increased differentiation and the localization of the tight junction and desmosome proteins. TEER can be affected by the molecular composition and organization of the tight junction, as this can also contribute to TEER [79,80]. It is possible that AZM causes altered molecular organization and/or localization of cell-cell junctional components, and that this change, in turn, leads to increased barrier integrity reflected in TEER and paracellular measurements [81].

Another factor we have not discussed fully is the possible effect of phospholipid retention on the epithelial barrier. AZM has a high affinity to lipids, as has been reported by a variety of authors, whereby it causes phospholipid retention by the binding of positively charged amino groups to negatively charged bilayers which, in turn, protects the phospholipids from degradation by phospholipases [40,82,83]. This ability of AZM treatment to increase phospholipid retention and vesicle formation has been reported previously in cellular and murine models [41,84]. This action may directly or indirectly contribute to the barrier-enhancing effects, as the endothelial barrier, for example, is enhanced by oxidized phospholipids but disrupted by truncated lipid products [85]. It is also possible that inhibition of degradation of phospholipids contributes to the therapeutic benefit of AZM in COPD, as fatty acid metabolism is disturbed during COPD [86]. In this connection, it is worth noting that the inflammatory cytokines interferon-γ and tumor necrosis factor-α, both disturb phospholipid composition and displace occludin from tight junctions in the T84 intestinal epithelial cell line, suggesting a process that might also be targeted by AZM in lung inflammation [87].

If phospholipid retention is a factor in barrier integrity, it may well explain the slight, tight junction protein-independent TEER increase we observe after treatment with CLARI and ERY, as they also significantly increased phospholipid accumulation, albeit not to the same degree as AZM treatment. It is of note that the vesicular buildup in AZM-treated samples was not seen in CLARI and ERY-treated samples.

Although macrolide antibiotics have similar antibacterial functions, it is clear they affect the human bronchial epithelium in distinct ways, and considerable work is needed to decipher the reasons for these differences. ERY and CLARI, the most closely structurally related compounds, cause similar physical changes but are not as distinct as those caused by AZM. Common to all compounds tested is the negative enrichment of some EMT pathway gene sets. Although not commonly recognized, EMT is a contributing factor to barrier failure [88]. In bronchial epithelium, cells undergoing EMT lose their mucociliary clearance properties and cell-cell adhesion, resulting in poor clearance and easier paracellular access for the infectious agents to the underlying submucosa. It is thus relevant that a common property among macrolides is their dampening effects on EMT. Indeed, AZM has been reported to suppress EMT mediated by TGF-β1 [89,90,91] and to reduce TGF-β-induced collagen production in fibroblasts from idiopathic pulmonary fibrosis (IPF) patients [92]. CLARI has been shown to likely attenuate EMT via the PI3K/Akt/mTOR signaling pathway [93]. Moreover, a deglycosylated variant of AZM has been shown to inhibit TGF-β1 signaling [94]. We observed that JAK-STAT, Notch, TGFβ, Hedgehog, and WNT signaling pathways were all affected negatively by the tested compounds but to varying degrees, and regulatory changes in these signaling pathways have all been shown to be involved in the disease process in COPD [95,96,97,98,99,100].

The effect of AZM on *COL1A2*, *COL3A1*, *COL5A2*, *MMP2*, and *MMP9* gene expression hints at its role in collagen remodeling. Increased collagen turnover is a marker of stable COPD and is elevated during exacerbations, suggesting that this may be one of the many avenues by which AZM asserts its off-label effects in COPD [101,102]. In IPF, excessive collagen production is prevalent as a result of an increased number of myofibroblasts. Furthermore, EMT is a prominent event in IPF [103].

## 4. Materials and Methods

### 4.1. Cell Culture

VA10 cells, a bronchial epithelial cell line immortalized by E6/E7 viral oncogenes, were cultured in Bronchial/Tracheal Epithelial Cell Growth Medium (511A-500, Cell Applications, San Diego, CA, USA). Cell line sourced from the lab that established them at the University of Iceland [6]. Cells were sub-cultured at a confluency of 70–80% and then seeded at a density of 200,000 cell/25 cm^2^. For ALI cultures, 200,000 cells were seeded on 12 mm Transwell membrane inserts with 0.4 µm pores (3460, Corning, Glendale, AZ, USA), and allowed to grow for 72 h in DMEM + 10% FBS to reach confluency. Then, the media was changed to Dulbecco’s Modified Eagle Medium (DMEM, Gibco, Paisley, UK) + 2% Ultroser G (15950-017, Sartorius, Cergy, France), and media was only added to the basal chamber to create the ALI condition. Treatment with drugs was then started at a concentration of 35 µM for each drug and with an equal volume of the vehicle control, to a final concentration of 0.1% dimethyl sulfoxide (DMSO) in each culture. Previous publications have used a range of concentrations from 31.8 to 53.4 µM [41,42,43]. All drugs were added to the media in the basal chamber. AZM (83905-01-5, Apollo Scientific, Manchester, UK), CLARI (A387-100MG, Sigma, St. Louis, MO, USA), ERY (E5389-1G, Sigma), ROXI (R4393-10G, Sigma), SOLI (A14031-5, Adooq Bioscience, Irvine, CA, USA), and TOBRA (T4014-100MG, Sigma).

### 4.2. Transepithelial Electrical Resistance

Transepithelial electrical resistance (TEER) was measured on days 0, 9, 14, and 21 of ALI cultures. Media at 37 °C was added to the apical chamber and incubated for 30 min at 37 °C before measuring TEER using a Millicell ERS-2 Voltohmmeter (MERS00002, Millipore, Burlington, MA, USA) to measure the electrical resistance across the culture.

### 4.3. Paracellular Flux

On day 21, the medium was changed to phenol-free and 1 mM Lucifer yellow CH dipotassium salt (L0144, Sigma-Aldrich, St. Louis, MO, USA) in Hanks’ Balanced Salt solution was added to the apical side and incubated for 2 h at 37 °C, 5% CO_2_. A total of 100 μL of the basolateral media was then collected and measured at excitation 485/emission 530 nm on a SpectraMax microplate reader.

### 4.4. RNA Sequencing and Analysis of Gene Expression

RNA from ALI cultures was isolated on days 14 and 21 using TRI Reagent^TM^ (AM9738, Invitrogen, Carlsbad, CA, USA) and sent to Qiagen Genomic Services where the RNA sequencing was performed.

Kallisto (v0.46.1) was used for quantifying RNA transcript expression, and the Sleuth R package v0.30 was used for gene expression estimation computing.

Gene set enrichment analysis (GSEA) was performed with the GSEA software v4.3.2 from a pre-ranked gene list, prepared by ordering genes (one transcript per gene) by expression difference significance (q-value multiplied with the sign of the log-fold change). Ontology gene sets and curated gene sets were used for the analysis. Principal component analysis (PCA) was performed using DESeq2 (v1.42.1).

### 4.5. Immunoblotting

Cells were lysed in radio-immunoprecipitation buffer with protease and phosphatase inhibitors. The cell lysate was then quantified using Bradford reagent (B6916, Sigma). Electrophoresis was performed on a NuPAGE 4 to 12% gel (NP0323BOX, Invitrogen) and resolved on PVDF membranes (Invitrogen).

PageRuler plus prestained protein ladder (26620, Thermo Scientific, Rockford, IL, USA) was used as a standard. The primary antibodies used were zonula occludens (ZO-1; Invitrogen, 33-9100), occludin (Invitrogen, 33-1500), desmoglein-1 (DSG-1; Proteintech, Manchester, UK, 24587-1-AP), caveolin-1 (CAV-1; Proteintech, 16447-1-AP), and glyceraldehyde-3-phosphate dehydrogenase (GAPDH; Proteintech, 10494-1-AP). Imaging was conducted using Odyssey Licor and ImageQuant, and Western blotting luminol reagent (sc-2048, Santa Cruz, Dallas, TX, USA) was used for chemiluminescence resolving. FIJI (ImageJ, v1.54i) was utilized for quantification.

### 4.6. Lipid Retention

VA10 cells were seeded and incubated overnight. Treatment and HCS LipidTOX^TM^ Green Phospholipidosis detection reagent (H34350, Invitrogen) were added to cultures and incubated for 72 h. Cells were then fixed in 4% paraformaldehyde (PFA) methanol-free (28906, Thermo Scientific) and nuclei were stained with 4′,6-diamidino-2-phenylindole (DAPI). Wells were then imaged and analyzed using a CellProfiler (v4.2.6, Broad Institute) pipeline, where cells were identified by the DAPI-stained nucleus, and the intensity of the stained phospholipids was quantified in relation to each cell [57].

### 4.7. Immunostaining

ALI cultures on Transwell filters were fixed in 4% PFA in PBS (Phosphate-Buffered Saline) before embedding in paraffin wax and cross-sectioned using a microtome. Sections were then stained using hematoxylin and eosin (H&E).

For imaging tight junctions, cultures were fixed in 1:1 (*v*/*v*) methanol and acetone at –20 °C. Samples were washed in tris-buffered saline before permeabilization with IMF (NaCl, HEPES, and EDTA) buffer, blocked with goat serum, and incubated with various primary antibodies, ZO-1 (Invitrogen, 33-9100), occludin (Invitrogen, 33-1500), claudin-4 (Invitrogen, 32-9400), DSG-1, (Proteintech, 24587-1-AP), junction plakoglobin (JUP; Proteintech, 66445-1-Ig), acetylated tubulin (Abcam, Cambridge, UK, ab24610), and cytokeratin 14 (CK14; Abcam, ab7800). Nuclei were visualized using DAPI. Stained samples were imaged on an Olympus FV1200 confocal microscope (Olympus, Tokyo, Japan), a Cicero Spinning Disk Confocal (CrestOptics, Rome, Italy), and an EVOS FL Auto 2 imaging system (ThermoFisher).

### 4.8. Transmission Electron Microscopy

VA10 cells were grown on Transwell filters and treated with macrolides as described previously. On day 21, they were fixed in 2% glutaraldehyde and 2% PFA in 0.1 M sodium-cacodylate buffer (NaCac) pH 7.4 at 4 °C for 2 h. Cells were then washed twice in 0.1 M NaCac pH 7.4 for 5 min, then osmicated with 1% OsO4 in 0.1 M NaCac pH 7.4 for 1 h on ice. Samples were again washed twice with 0.1 M NaCac pH 7.4 for 5 min and dehydrated using an ethanol gradient and acetone before embedding in Epon and baking for 18 h at 6 °C. Cells were then embedded in resin, sectioned with an ultramicrotome (Leica EM UC7, Wetzlar, Germany), and imaged on a JEM-1400PLUS PL transmission electron microscope.

### 4.9. Venn Diagrams

Venn diagrams [104] were made by using the same ranked gene lists used for GSEA, but were separated by whether they were positively or negatively regulated when compared to the control samples. Groups of genes from Venn groupings were then further analyzed by PANTHER 18.0 by performing a statistical overrepresentation test for gene ontology.

### 4.10. Statistical Analysis

Statistical analyses of TEER, paracellular flux, and fluorescent intensity were carried out using GraphPad Prism version 9.4.1 for Windows (GraphPad Software, Boston, MA USA, www.graphpad.com). Statistical significance was established using one-way or two-way ANOVA where applicable. The threshold significance level was set at 0.05.

RNA sequencing data were quality checked and normalized for differential expressed genes (DEG) testing in the Sleuth package in R. Differential expression of transcripts and *p*- and q-values were calculated using the Sleuth R package (v0.30) on the normalized expression data, using the Kallisto (v0.46.1) bootstrapping values to estimate variance in pseudo alignments. For the GSEA analysis, no significance threshold is used. All genes/transcripts are given a ranking value from the negative logarithm of the q-value, and the sign of the ranking is decided by the fold change being negative or positive. The GSEA program (v4.3.2) uses these ranking values to estimate gene set enrichment.

## 5. Conclusions

In this study, we have presented possible factors that may contribute to the effect AZM exerts on bronchial epithelial barrier integrity. We propose that epithelial cell differentiation, maintenance of polarity, localization, and organization of cell-cell junctions, downregulation of EMT, CAV-1, and lipid accumulation may together be the reasons for the increase in epithelial integrity observed with AZM treatment. Further research is needed to elucidate if and how AZM affects these factors, and in what way they contribute to epithelial integrity.

## Data Availability

The original RNA sequencing data presented in the study were deposited at the Gene Expression Omnibus database (GEO) under accession number GSE278622 and will be openly available at the following URL: https://www.ncbi.nlm.nih.gov/geo/query/acc.cgi?acc=GSE278622 (accessed on 26 February 2025).

## Supplementary Materials

The following supporting information can be downloaded at: https://www.mdpi.com/article/10.3390/ijms26052287/s1.

## Abbreviations

The following abbreviations are used in this manuscript:

ALI	Air-liquid interface
AZM	Azithromycin
CAV-1	Caveolin-1
CF	Cystic fibrosis
CK14	Cytokeratin 14
CLARI	Clarithromycin
COPD	Chronic obstructive pulmonary disease
Ctrl	Control
DAPI	4′,6-diamidino-2-phenylindole
DMEM	Dulbecco’s Modified Eagle Medium
DMSO	Dimethyl sulfoxide
DSG-1	Desmoglein-1
EMT	Epithelial-to-mesenchymal transition
ERY	Erythromycin
GAPDH	Glyceraldehyde-3-phosphate dehydrogenase
GOCC	Gene ontology cellular component
GOBP	Gene ontology biological process
GSEA	Gene set enrichment analysis
H&E	Hematoxylin and eosin
IPF	Idiopathic pulmonary fibrosis
KEGG	Kyoto Encyclopedia of Genes and Genomes
LPS	Lipopolysaccharide
NaCac	Sodium-cacodylate buffer
PCA	Principal component analysis
PFA	Paraformaldehyde
ROXI	Roxithromycin
SOLI	Solithromycin
TEER	Transepithelial electrical resistance
TOBRA	Tobramycin
ZO-1	Zonula occludens-1
