# Supplementary material for: The Non-Antibacterial Effects of Azithromycin and Other Macrolides on the Bronchial Epithelial Barrier and Cellular Differentiation
Source: Int J Mol Sci. 2025 Mar 4;26(5):2287. doi: 10.3390/ijms26052287 (PMC11900332; doi:10.3390/ijms26052287)
Supplement: Supplementary file 1 [file ijms-26-02287-s001.zip › Supplementary Materials.pdf]

Supplementary Materials

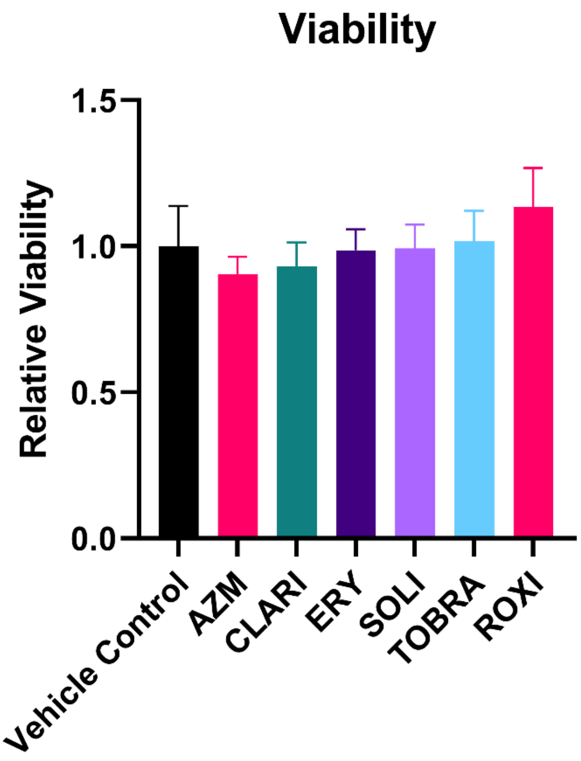

Figure S1. Macrolides do not affect the viability of VA10 Cells in monolayer cultures.
Graph showing the relative viability of VA10 cells when treated with macrolides at 35 uM in DMSO in monolayer. Viability was assessed using PrestoBlue cell viability reagent, and measured on a microplate reader.

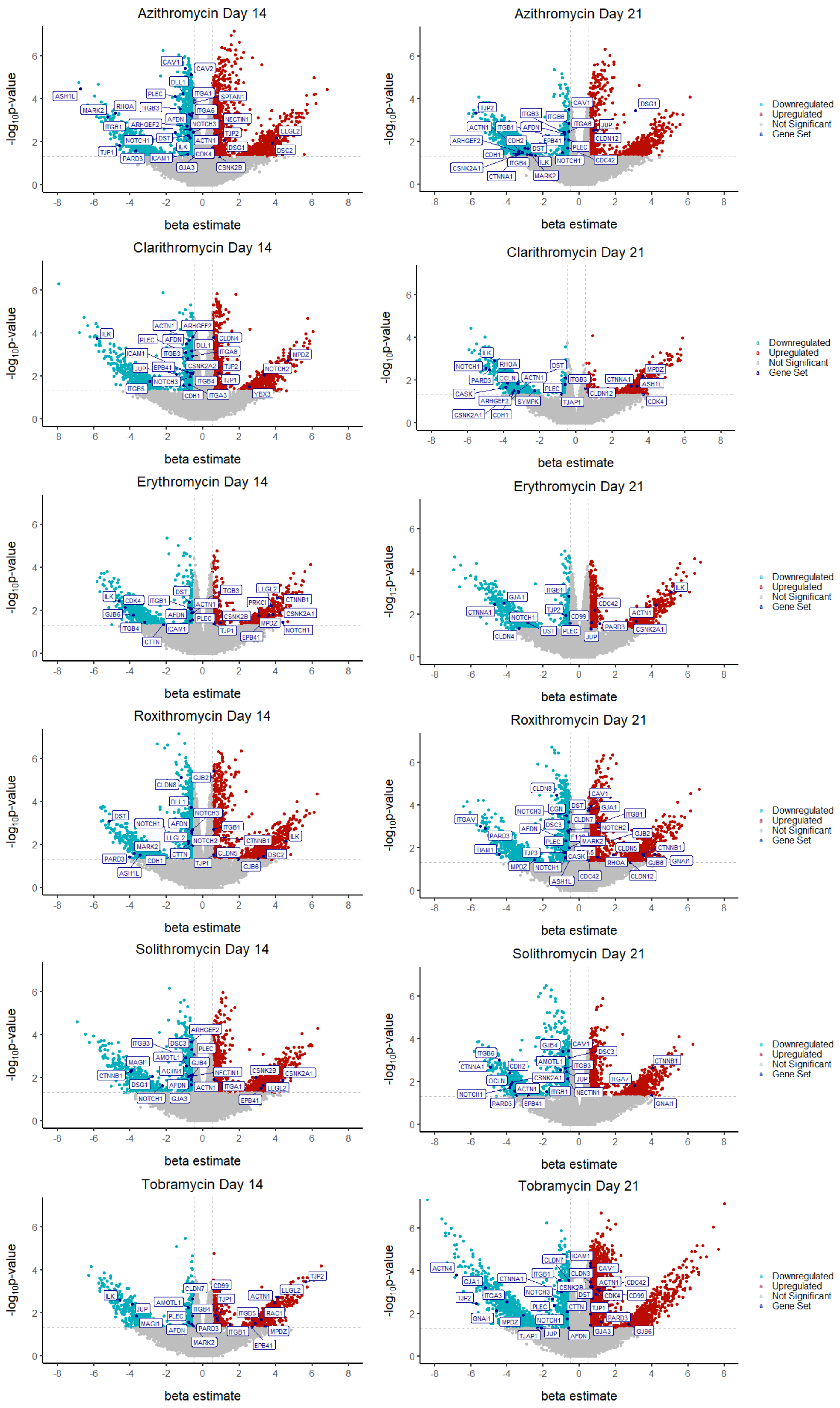

Azithromycin Day 14
Azithromycin Day 21
Clarithromycin Day 14
Clarithromycin Day 21
Erythromycin Day 14
Erythromycin Day 21
Roxithromycin Day 14
Roxithromycin Day 21
Solithromycin Day 14
Solithromycin Day 21
Tobramycin Day 14
Tobramycin Day 21
-log10p-value
beta estimate
Downregulated
Upregulated
Not Significant
Gene Set

Figure S2. Macrolides affect the expression of a variety of cell junction related genes

Volcanoplot highlighting focal adhesion, tight junction, gap junction adherens junction, desmosomal and hemidesmosomal genes that are significantly affected by treatment. Grey data points are either not significant (p-value > 0.05) or have a beta value lower than 0.05. Red color represents increased enrichment relative to control and blue represents negative enrichment relative to control.

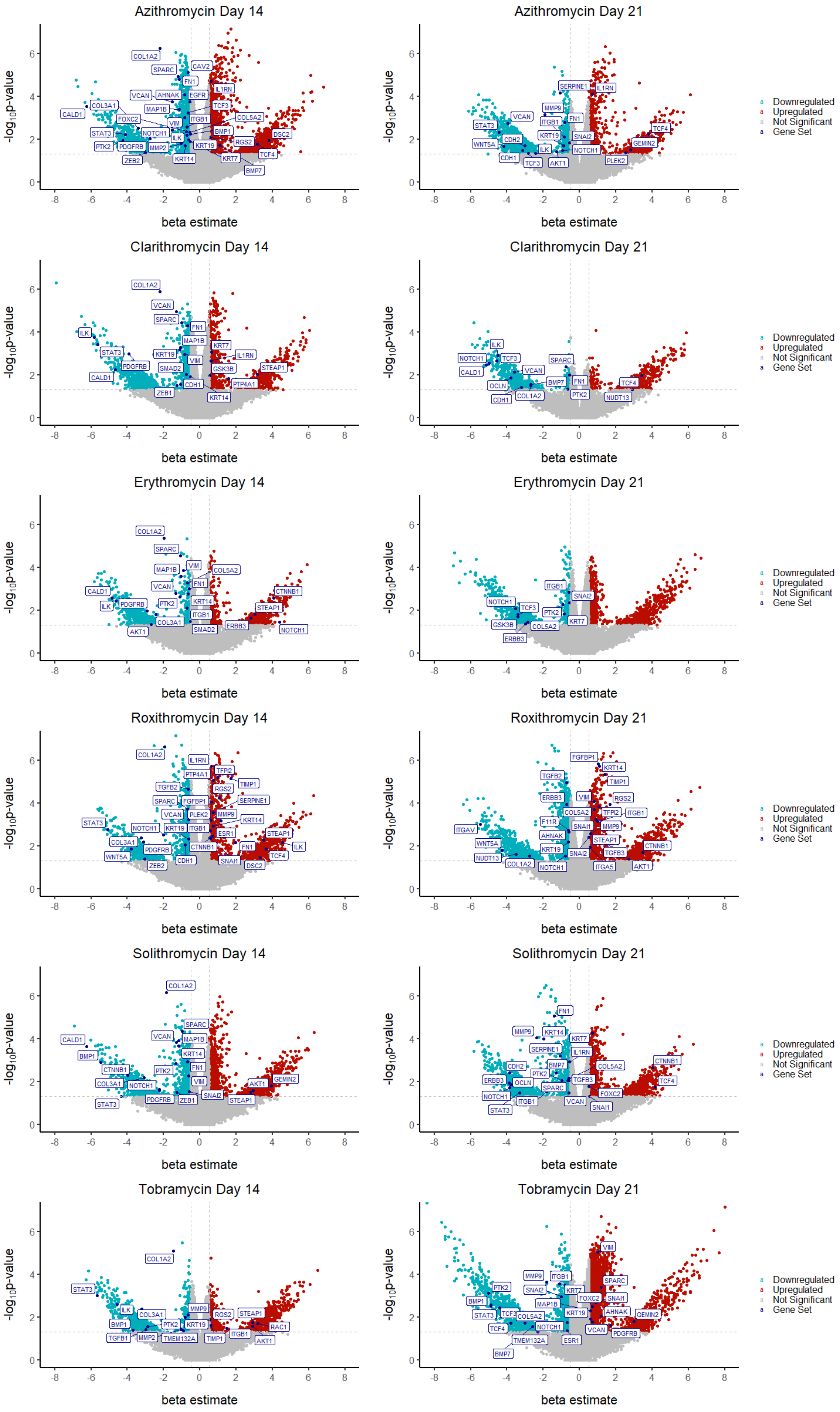

Azithromycin Day 14
COL1A2
SPARC
CAV2
FN1
IL1RN
VCAN
AHNAK
EGFR
COL3A1
CALD1
MAP1B
TCF3
FOXC2
VIM
ITGB1
COL5A2
STAT3
NOTCH1
BMP1
ILK
DSC2
PTK2
PDGFRB
MMP2
KRT19
RGS2
TCF4
ZEB2
KRT14
KRT7
BMP7
Azithromycin Day 21
SERPINE1
IL1RN
MMP9
VCAN
FN1
STAT3
ITGB1
TCF4
KRT19
SNAI2
WNT5A
CDH2
GEMIN2
ILK
NOTCH1
CDH1
PLEK2
TCF3
AKT1
Clarithromycin Day 14
COL1A2
VCAN
SPARC
FN1
ILK
MAP1B
KRT7
STAT3
KRT19
IL1RN
VIM
PDGFRB
SMAD2
GSK3B
STEAP1
CALD1
PTP4A1
CDH1
ZEB1
KRT14
Clarithromycin Day 21
ILK
NOTCH1
TCF3
SPARC
CALD1
VCAN
OCLN
BMP7
FN1
TCF4
CDH1
COL1A2
PTK2
NUDT13
Erythromycin Day 14
COL1A2
SPARC
MAP1B
VIM
COL5A2
VCAN
FN1
CALD1
CTNNB1
ILK
PDGFRB
PTK2
KRT14
STEAP1
ITGB1
COL3A1
ERBB3
AKT1
SMAD2
NOTCH1
Erythromycin Day 21
ITGB1
SNAI2
NOTCH1
TCF3
PTK2
KRT7
GSK3B
COL5A2
ERBB3
Roxithromycin Day 14
COL1A2
IL1RN
PTP4A1
TFPI2
TIMP1
TGFB2
RGS2
SPARC
FGFBP1
SERPINE1
VCAN
PLEK2
MMP9
STAT3
NOTCH1
KRT19
ITGB1
KRT14
ESR1
STEAP1
COL3A1
CTNNB1
FN1
ILK
PDGFRB
WNT5A
CDH1
SNAI1
TCF4
ZEB2
DSC2
Roxithromycin Day 21
FGFBP1
KRT14
TGFB2
TIMP1
ERBB3
VIM
RGS2
COL5A2
TFPI2
ITGB1
F11R
SNAI1
MMP9
ITGAV
AHNAK
STEAP1
WNT5A
KRT19
TGFB3
CTNNB1
SNAI2
NUDT13
COL1A2
NOTCH1
ITGA5
AKT1
Solithromycin Day 14
COL1A2
SPARC
VCAN
MAP1B
CALD1
KRT14
BMP1
CTNNB1
PTK2
FN1
COL3A1
NOTCH1
VIM
GEMIN2
AKT1
SNAI2
PDGFRB
ZEB1
STEAP1
STAT3
Solithromycin Day 21
FN1
MMP9
KRT14
KRT7
SERPINE1
IL1RN
CDH2
BMP7
COL5A2
CTNNB1
PTK2
ERBB3
OCLN
TGFB3
TCF4
SPARC
NOTCH1
FOXC2
ITGB1
VCAN
SNAI1
STAT3
Tobramycin Day 14
COL1A2
STAT3
ILK
COL3A1
MMP9
STEAP1
BMP1
PTK2
KRT19
RGS2
RAC1
TGFB1
MMP2
TMEM132A
TIMP1
ITGB1
AKT1
Tobramycin Day 21
VIM
MMP9
ITGB1
SPARC
PTK2
SNAI2
KRT7
FOXC2
SNAI1
BMP1
MAP1B
AHNAK
STAT3
TCF3
COL5A2
KRT19
GEMIN2
TCF4
NOTCH1
VCAN
PDGFRB
TMEM132A
ESR1
BMP7
-log10p-value
beta estimate
Downregulated
Upregulated
Not Significant
Gene Set

Figure S3. Macrolides affect the expression of a variety of EMT related genes. Volcanoplot highlighting EMT related genes that are significantly affected by treatment. Grey data points are either not significant (p-value > 0.05) or have a beta value lower than 0.05. Red color represents increased enrichment relative to control and blue represents negative enrichment relative to control.

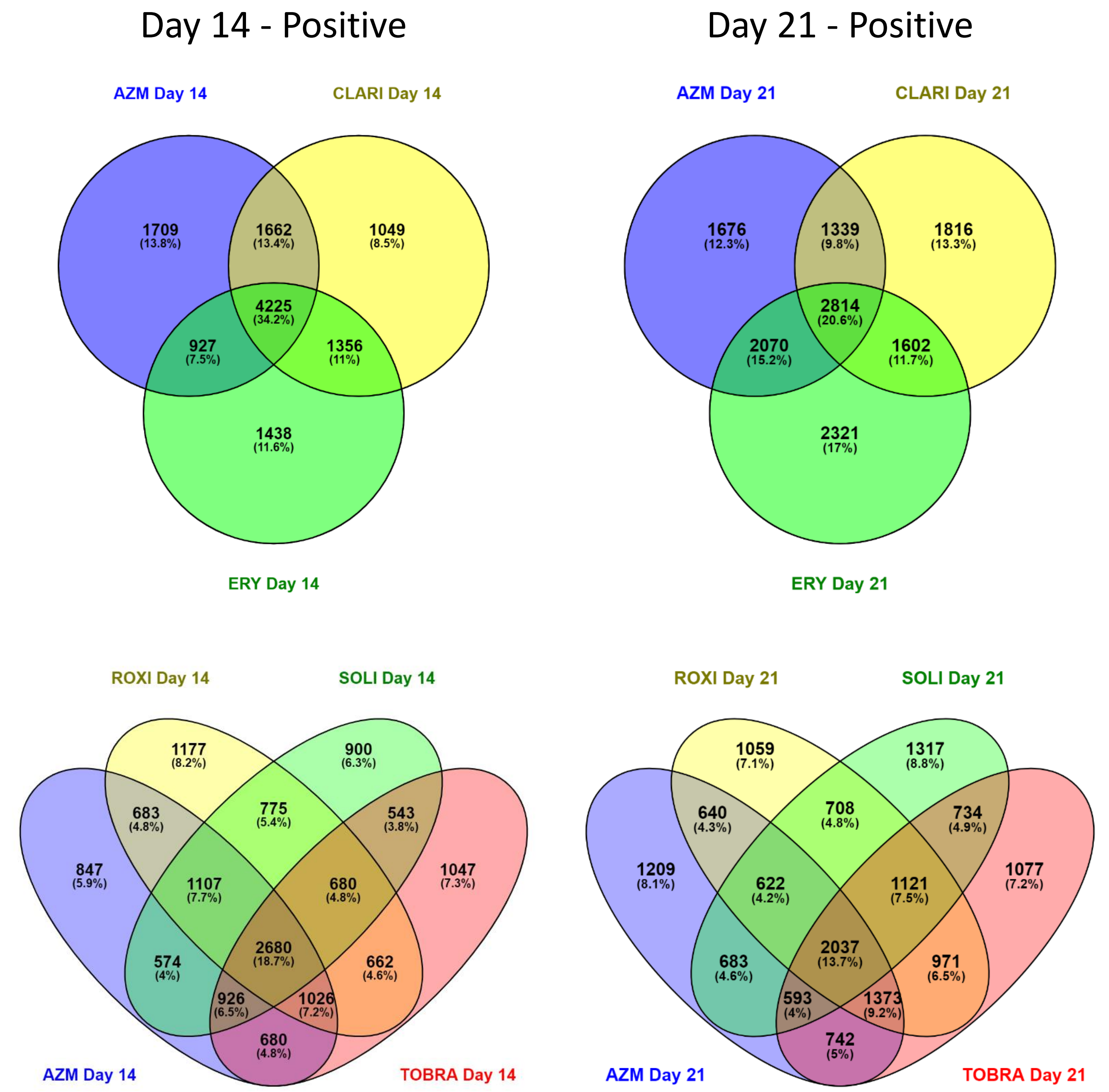

Figure S4. RNA sequencing of VA10 cells in ALI cultures demonstrate increase in gene expression differences over time in ALI

Venn diagrams comparing genes increased expression with macrolide treatment, at day 14 and 21, when compared to the vehicle control. For ease of viewing AZM comparison to other treatments is shown separately versus; CLARI and ERY, and ROXI, SOLI and TOBRA.

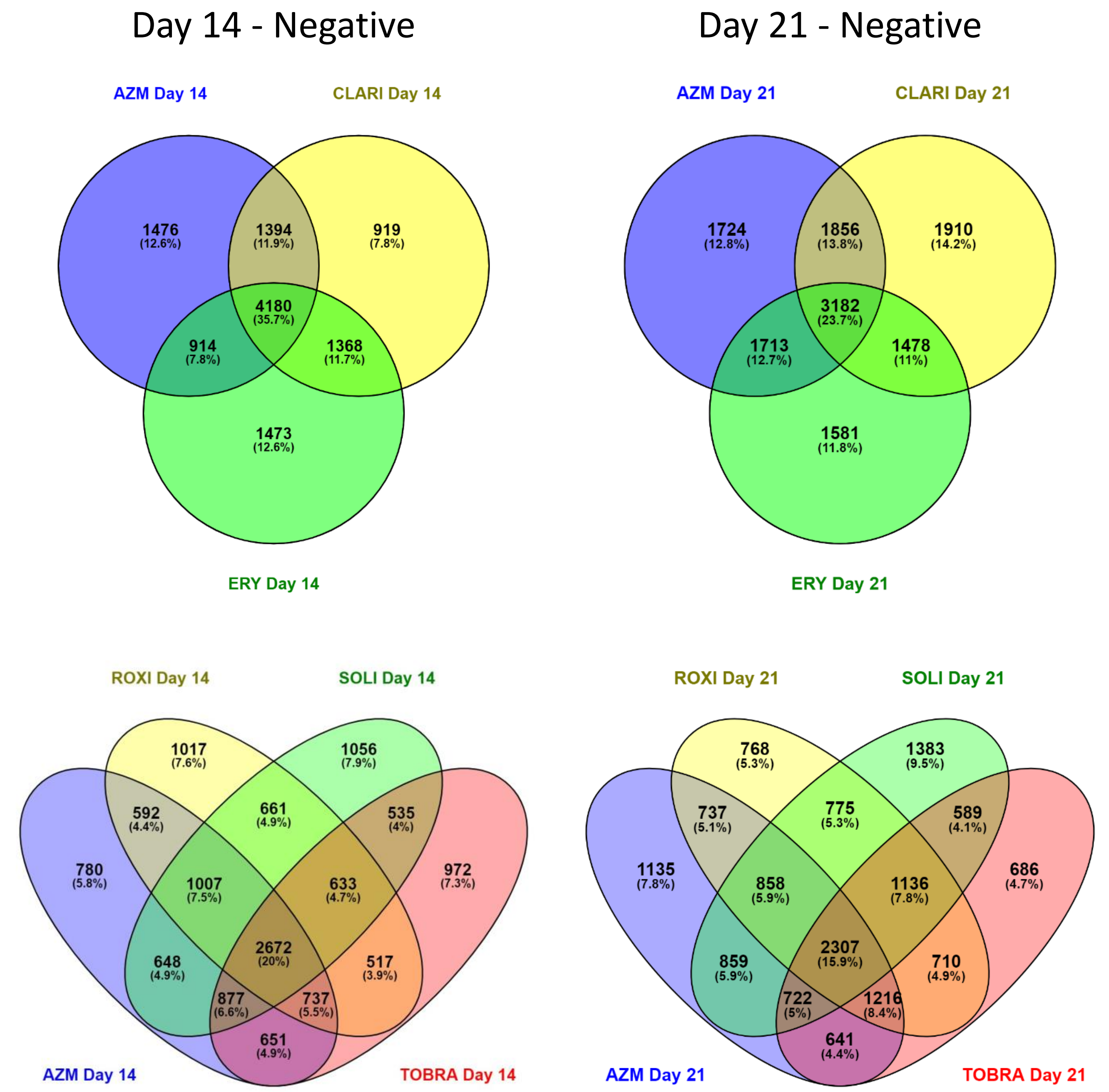

Figure S5. RNA sequencing of VA10 cells in ALI cultures demonstrate increase in gene expression differences over time in ALI
Venn diagrams comparing genes decreased expression with macrolide treatment, at day 14 and 21, when compared to the vehicle control. For ease of viewing AZM comparison to other treatments is shown separately versus; CLARI and ERY, and ROXI, SOLI and TOBRA.

Table S1. AZM uniquely affects a variety of genes.
PANTHER classification Overrepresentation test of a list of genes uniquely up- and downregulated with AZM treatment in ALI cultures on day 21. AZM shows unique Up-regulation of genes related to epidermis and skin development, endosome and vesicle components.

GO Sub-Ontology	Category	GO class
**GO biological process complete**	Up-regulated	epidermis development (GO:0008544)<br>skin development (GO:0043588)
	Down-regulated	cytoplasmic translation (GO:0002181)<br>ribosomal small subunit biogenesis (GO:0042274)<br>translation (GO:0006412)<br>peptide biosynthetic process (GO:0043043)<br>amide biosynthetic process (GO:0043604)<br>peptide metabolic process (GO:0006518)<br>ribosome biogenesis (GO:0042254)<br>ribonucleoprotein complex biogenesis (GO:0022613)<br>amide metabolic process (GO:0043603)<br>organonitrogen compound biosynthetic process (GO:1901566)<br>RNA processing (GO:0006396)<br>gene expression (GO:0010467)<br>macromolecule biosynthetic process (GO:0009059)<br>cellular nitrogen compound metabolic process (GO:0034641)<br>cellular nitrogen compound biosynthetic process (GO:0044271)<br>nucleobase-containing compound metabolic process (GO:0006139)<br>cellular biosynthetic process (GO:0044249)<br>organic substance biosynthetic process (GO:1901576)<br>heterocycle metabolic process (GO:0046483)<br>Cellular aromatic compound metabolic process (GO:0006725)<br>nucleic acid metabolic process (GO:0090304)<br>biosynthetic process (GO:0009058)<br>organic cyclic compound metabolic process (GO:1901360)
**GO cellular component complete**	Up-regulated	cornified envelope (GO:0001533)<br>azurophil granule membrane (GO:0035577)<br>endosome membrane (GO:0010008)<br>endosome (GO:0005768)<br>vesicle membrane (GO:0012506)<br>cytoplasmic vesicle membrane (GO:0030659)<br>cytoplasmic vesicle (GO:0031410)<br>intracellular vesicle (GO:0097708)<br>vesicle (GO:0031982)<br>endomembrane system (GO:0012505)<br>cytoplasm (GO:0005737)
